# Assessment of Oral Hygiene Behavioral and Demographic Risk Factors for Extrahepatic Manifestations of Hepatitis C

**DOI:** 10.3390/medsci13040298

**Published:** 2025-12-03

**Authors:** Mihai Oprea, Andreea Cândea, Alexandra Roman, Ion Rogoveanu, Allma Roxana Pitru, Claudiu Marinel Ionele, Dorin Nicolae Gheorghe, Flavia Mirela Nicolae, Dora Maria Popescu, Adina Turcu-Stiolica, Sergiu Ciobanu, Petra Surlin

**Affiliations:** 1 Department of Periodontology, Doctoral School, Faculty of Dental Medicine, University of Medicine and Pharmacy of Craiova, 200349 Craiova, Romania; 2 Department of Periodontology, Faculty of Dental Medicine, Iuliu Hatieganu University of Medicine and Pharmacy, 400012 Cluj-Napoca, Romania; 3 Department of Gastroenterology, Faculty of Medicine, University of Medicine and Pharmacy of Craiova, 200349 Craiova, Romania; 4 Department of Oral Pathology, Research Center of Periodontal-Systemic Interactions, Faculty of Dental Medicine, University of Medicine and Pharmacy of Craiova, 200349 Craiova, Romania; 5 Department of Periodontology, Research Center of Periodontal-Systemic Interactions, Faculty of Dental Medicine, University of Medicine and Pharmacy of Craiova, 200349 Craiova, Romania; 6 Department of Pharmacoeconomics and Statistical Analysis, Faculty of Pharmacy, University of Medicine and Pharmacy of Craiova, 200349 Craiova, Romania; 7 Department of Odontology, Periodontology and Oral Pathology, “Nicolae Testemitanu” State University of Medicine and Pharmacy of the Republic of Moldova, MD-2004 Chisinau, Moldova

**Keywords:** extrahepatic manifestations, HC, smoking, oral hygiene behavior, xerostomia, OLP

## Abstract

**Background:** Hepatitis C (HC) remains a major public health concern, affecting approximately 50 million people globally. In addition to hepatic damage, HC induces extrahepatic manifestations (EHMs), including oral conditions such as oral lichen planus (OLP), xerostomia, and Sjögren syndrome-like (SS-like), which impair quality of life. The aim of this study was to investigate the possible association between certain extrahepatic manifestations of HC and the presence of risk factors. **Methods:** A cross-sectional study was conducted on 38 adults (22 males and 16 females; mean age 56.5 ± 8.6 years) with inactive HC. For each patient, demographic and clinical data were collected, including the following: frequency of dental brushing, frequency of professional dental hygiene visits, smoking, alcohol consumption, the presence of xerostomia, OLP, and SS-like. Logistic regression analyses and ROC curves were performed using R software to identify independent predictors for each condition. **Results:** OLP was present in 39.5%, xerostomia in 47.4%, and SS-like in 15.8% of patients. Female gender significantly predicted OLP and showed a borderline association with xerostomia. Smoking was weakly associated with xerostomia. No predictors were significant for SS-like. **Conclusions:** Oral hygiene and smoking are risk factors for oral EHM, their good control being important for the quality of life of these patients. Gender has also been shown to be a risk factor for these manifestations.

## 1. Introduction

Despite efforts that have been made in the past few years to enhance the prevention and implement treatments, hepatitis C (HC) remains an important global health issue. An estimated 50 million people worldwide still live with HC [1], which is a decrease compared to the 71 million reported in 2015 [2]. The untreated viral infection causes long-term chronic inflammation of the liver, leading to complications such as hepatic cirrhosis and hepatocellular carcinoma that are fatal for the patient [3]. Moreover, the pathological events that hepatitis C virus (HCV) infection triggers expand beyond the liver as extra-hepatic manifestations (EHMs), including oral ones like oral lichen planus or xerostomia, affecting the quality of life of these patients [4,5]. Periodontal disease was also included among the EHMs of HC infection [4], making the reduction in periopathogen load and, consecutively, the maintenance of good oral hygiene, important treatment goals for patients with HC, including those with EHMs. Thus, oral hygiene is considered a risk factor for these patients [4].

According to a WHO report, in Romania, 136,999 persons were living with chronic hepatitis C in 2022 [6], representing 0.7% of the general population [7]; this was a decline compared to the 550,000 (2.7%) people reported to be living with the condition in 2015 [2].

Oral lichen planus is a chronic inflammatory disorder of the oral mucosa that could be associated with systemic diseases such as HC [4], but recent research suggests a bidirectional relationship that should be taken into consideration when conducting screening in countries with an increased prevalence of HCV among the general population [8].

Xerostomia, the sensation of oral dryness, could be caused by various etiologies like systemic diseases and conditions, medications, and lifestyle habits like alcohol and tobacco consumption with a higher prevalence in women [9] and it has been reported to have variable prevalence in HCV-infected individuals [10,11].

Sjögren syndrome (SS) is a recognized autoimmune disease characterized by xerostomia (the salivary glands being infiltrated by a wide spectrum of immune cells) [4,9], as well as eye dryness, joint pain, and fatigue in approximately 80% of patients, alongside bilateral swelling of parotid glands and swelling joints [12]. The 2016 American–European Consensus Criteria for the classification of primary SS determined that evidence of HCV infection is an exclusion criterion for the classification of a patient as having SS [13], suggesting that HCV is directly involved in the modifications of salivary glands, but the exact pathogenic mechanism driving the xerostomia has not been elucidated [14]. HCV patients could manifest symptoms similar to SS (SS-like), which are considered as extrahepatic manifestations of this viral infection [14].

The *aim* of this study was to investigate the possible association between certain extrahepatic manifestations of HC and the presence of risk factors such as demographic, smoking and oral hygiene.

## 2. Materials and Methods

### 2.1. Study Subjects

The current study was performed on a sample of 38 patients (22 males and 16 females) aged 38–67 years with inactive chronic HC monitored by the Department of Gastroenterology—the County Hospital of Emergency of Craiova, University of Medicine and Pharmacy of Craiova, Romania. The ethics approval for this study was obtained from the research Ethic Commissions within the University of Medicine and Pharmacy of Craiova and Clinical County Hospital of Emergency of Craiova. The inclusion criteria were as follows: adult patients, both men and women, diagnosed with inactive chronic hepatitis C. The exclusion criteria encompassed patients who had been diagnosed with (and received treatment for) other diseases, as well as pregnant women.

This study was carried out between March and October 2024 in full accordance with the World Medical Association Declaration of Helsinki and all the patients included in this study signed an informed consent form.

### 2.2. Patients Examination

The patients underwent anamnestic and clinical oral examination by a well-trained dentist (M.O). For each patient, demographic (gender, age) and clinical data were collected from the chart, including the following: frequency of dental brushing; frequency of professional dental hygiene visits; smoking; the presence of xerostomia (the sensation of dry mouth, self-reported, without rating its severity or measuring the saliva flow); clinical appearance of oral lichen planus (OLP), regardless of its type or oral distribution; and SS-like symptoms. Regarding alcohol consumption, patients reported an intake within the limits of the national low-risk drinking recommendations for Romania, published by the European Commission; as a result, the alcohol intake was not included in the predictors [15].

The frequency of dental brushing was registered as follows: less than daily, once per day, twice per day. Dental hygiene visits frequency was assessed according to the following categories: the last visit took place more than 1 year ago; the last visit took place within the past year; or the last visit took place within the last 6 months. For smoking, the patients were separated into the following categories: non-smokers, smokers who smoked less than 10 cigarettes/day, and smokers who smoked more than 10 cigarettes/day, using the same threshold to record the periodontal risk or modify the periodontitis grade [16]. The patients were recorded as having SS-like if they presented at least two of the following clinical manifestations: eye dryness, joint pain, joint swelling, fatigue, and swelling parotids. In all patients who presented clinical lesions of OLP, a biopsy was performed and the histopathological exam confirmed the diagnosis. The presence of xerostomia was recorded separately.

### 2.3. Statistical Analysis

We performed descriptive statistical calculations of categorical variables (gender, smoking, frequency of brushing, professional oral hygiene, OLP, xerostomia, and Sjögren syndrome-like) with counts and percentages, and continuous variables (age) with mean ± standard deviation (SD), median (interquartile range, IQR), and range.

Separate binary logistic regression for each condition (OLP, xerostomia, and Sjögren syndrome-like) was performed to predict them using all predictors (gender, age, brush, hygiene, and smoking) simultaneously and to identify which variables are independent predictors of each one after adjusting for others. VIF > 10 indicates severe collinearity. We further wanted to test if these predictors influence the joint occurrence of OLP, xerostomia, and Sjogren syndrome-like. The alluvial diagram visually explores the interrelatedness of OLP, xerostomia, and Sjögren syndrome within the study group. Statistical analyses were performed using R software for Windows (version 4.3.0; R Core Team, Vienna, Austria, 2023) with the packages multipleROC (Moon, K 2020. Multiple ROC: Package for ROC analysis with models with multiple predictors), and easyalluvial (Bjoern Koneswarakantha 2019. Easyalluvial: Generate Alluvial Plots with a Single Line of Code. Retrieved from https://cran.r-project.org/web/packages/easyalluvial/index.html; accessed on 15 June 2025). We performed post hoc computation using G*Power 3.1.9.7. The post hoc analysis revealed that the study achieved 76% power in the detection of medium–large effects (f^2^ = 0.33).

## 3. Results

### 3.1. General Characteristics of Study Participants

The study group included 38 patients with ages ranging from 38 years to 67 years and an average age of 56.5 ± 8.6 years. The gender breakdown was 58% male and 42% female. Twenty-six percent were non-smokers, forty-two percent smoked less than 10 cigarettes/day, and thirty-two percent smoked more than 10 cigarettes/day, as shown in Table 1. Most of the patients had xerostomia—approximately 50% (47.4).

The most prevalent teeth brushing habit was daily (57.9%) and, regarding the professional hygiene behavior, the most prevalent visits were at 1 year (60.5%).

### 3.2. Prevalence of Oral Hygiene Habits in Patients with EHC Manifestations

Most patients with EHM-assessed manifestations reported brushing daily, whilst brushing twice per day was reported by the fewest patients. Most patients with xerostomia and OLP reported having visited a dental hygienist within the past year, and less than 6 months prior for patients with SS-like. Going more than 1 year between dental hygiene checkups was the least commonly reported behavior associated with each EHM (Table 2).

### 3.3. Logistic Regression for OLP

Gender (female) was found to be the strongest predictor of OLP (OR = 10.61, 95% CI: 1.78–94.45), with females having 10.6× higher odds of OLP than males (*p* < 0.05). No significant impact was found for the other factors, as shown in Table 1. No multicollinearity was observed: all VIFs were less than 5, as shown in Table 3.

**Figure 1:**
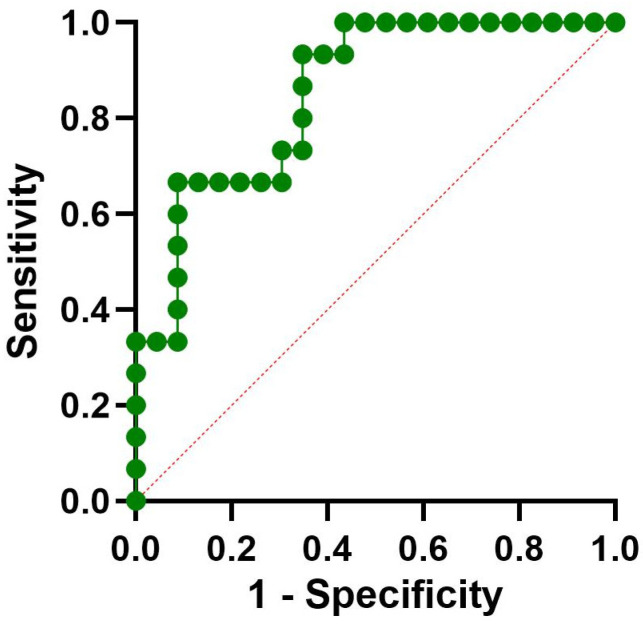
ROC curve assessing the performance of the logistic regression model used to predict OLP.

The model performance showed good discrimination: the area under the ROC was 0.8522 (95% CI: 0.73–0.97, *p* = 0.0003), as shown in Figure 1. An AUC > 0.8 is generally considered excellent, indicating that the model reliably distinguishes between patients with and without OLP. The narrow confidence interval around the AUC (despite the wide OR CI) further supports the robustness of the findings.

### 3.4. Logistic Regression for Xerostomia

Slightly higher odds for the limit of statistical significance were observed for female gender (OR = 2.18, 95% CI: 0.43–12.17, *p* = 0.035), as shown in Table 4. The trend toward higher xerostomia odds, which was also on the border of significance, in smokers suggested that tobacco may exacerbate dry mouth (OR = 1.73, CI 95%: 0.14–28.14, *p* = 0.057). No other variables (age, brushing, hygiene, smoking) showed statistically significant associations with xerostomia (all *p*-values > 0.05)

**Figure 2:**
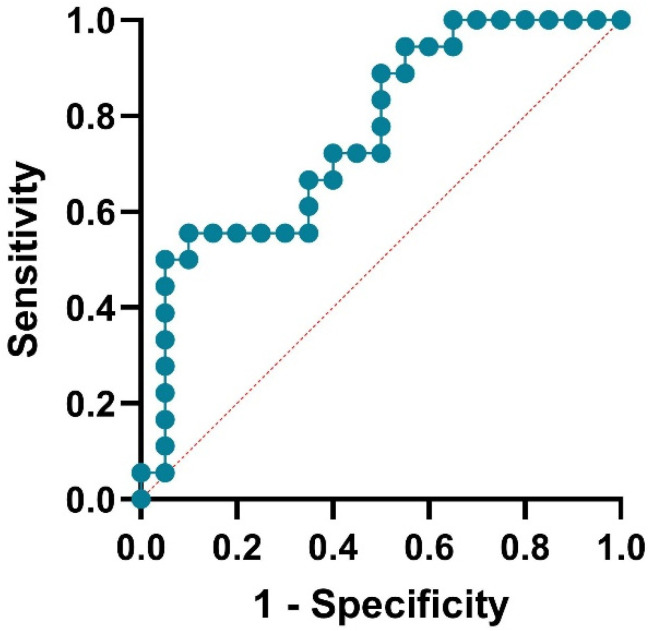
ROC curve assessing the performance of the logistic regression model used to predict xerostomia.

The model performance showed moderate discrimination: The area under the ROC was 0.7611 (95% CI: 0.61–0.91, *p* = 0.0060), as shown in Figure 2. No evidence of poor fit was observed (Hosmer–Lemeshow *p* = 0.33). An AUC of 0.7–0.8 is generally considered acceptable but suggests room for improvement. The statistically significant AUC (*p* < 0.01) implies the model outperforms chance, though its clinical utility may be limited without stronger predictors.

### 3.5. Logistic Regression for Sjögren Syndrome-like

We could not fit a logistic regression including all predictors. This could have been due to perfect separation, or because one or more of the predictors were linearly dependent. Our model included gender, age, and brushing as predictors, but none of these showed a statistically significant association with Sjögren syndrome-like, as shown in Table 5.

**Figure 3:**
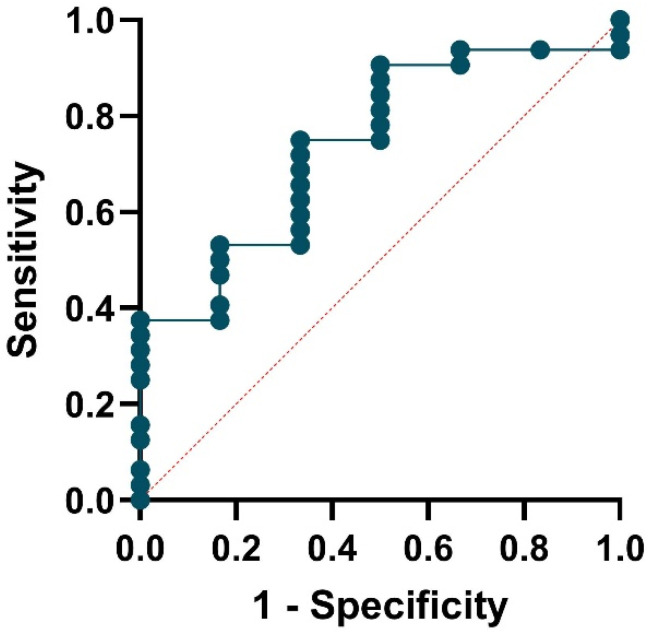
ROC curve assessing the performance of the logistic regression model used to predict the Sjögren syndrome-like.

The model’s performance showed moderate discrimination ability. The area under the ROC was 0.74 (95% CI: 0.54–0.94, *p* = 0.0656), as shown in Figure 3. No evidence of poor fit was observed (Hosmer–Lemeshow *p* = 0.59).

### 3.6. Alluvial Diagram Analysis for OLP, Xerostomia, and Sjögren Syndrome-like

**Figure 4:**
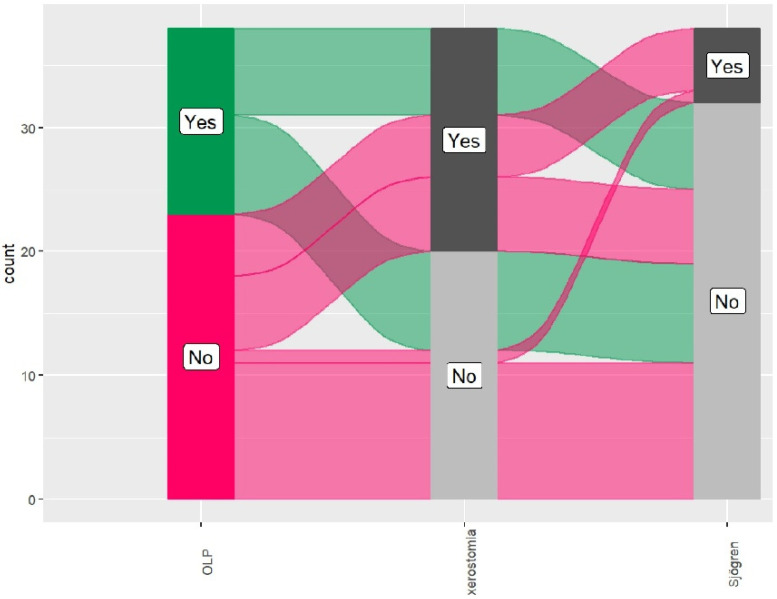
Alluvial diagram analysis of the interconnectedness of OLP, xerostomia, and Sjögren syndrome-like.

The alluvial diagram illustrates the interconnectedness of the three conditions across the study group, as shown in Figure 4. The diagram depicts six distinct flows, representing unique pathways or combinations of the analyzed variables (e.g., transitions between “Yes” and “No” states for the three conditions—OLP, xerostomia, or Sjögren syndrome-like). The color coding in this alluvial plot is designed to trace specific patient groups based on their initial diagnosis, treating OLP status as the primary stratifying variable. The green ribbons represent the trajectory of patients positive for OLP (“Yes”), while the magenta ribbons track those without the condition (“No”). By maintaining these specific hues across the entire flow, the chart allows to visually isolate the comorbidities associated with each starting group; for instance, the dominance of green ribbons entering the “Yes” node for Sjögren’s reveals that nearly all patients with this syndrome in the dataset originated from the OLP-positive group. The vertical grey bars serve as neutral aggregation nodes—dark grey for the presence of a condition and light grey for its absence—acting as checkpoints to quantify how the two original cohorts distribute themselves across the variables of xerostomia and Sjögren’s syndrome. 

The predominant pattern was indicated as the maximum weight of a single flow accounting for 28.9% of cases, representing patients without OLP, without xerostomia, and without Sjögren syndrome. No flow was observed, for all three conditions coexist. No co-occurrence of OLP and Sjögren syndrome was observed. Xerostomia evidenced a bridge between OLP (18.4% of patients in the study group had OLP and xerostomia, suggesting that the latter may arise as a result of secondary salivary dysfunction caused by OLP) and SS-like (13.2% of patients in the study group had both xerostomia and SS-like).

## 4. Discussion

In our study, the most prevalent gender was male (57.9%) according to the literature data, which revealed that HCV RNA positivity is significantly higher in males than females in adults, while there are no gender differences in children [17]. We identified an epidemiological recognized ratio of 1.6:1 males–females [18], especially in men who have sex with men (MSM) [19].

Our results showed a higher percentage of patients reporting xerostomia, followed by OLP and SS-like (47.4/39.5/15.8%), consistent with the literature reporting this association in up to 55% of patients with HCV [14].

The results of Maldonado et al. [14] suggest a novel mechanism distinct from SS that may explain the xerostomia in the HCV patients; furthermore, it supports a role for virus infection in salivary gland dysfunction, with the inflammation being driven by the presence of HCV RNA in the glands, as evidenced by increased numbers of CD8+ T cells. The authors stated that further studies will be needed to support these findings and better understand the molecular mechanisms associated with HCV infection and xerostomia.

We found that the presence of xerostomia was associated with female gender in accordance with literature data [20] and with the increased number of cigarettes [21]. According to Guo et al. [21], e-cigarette users were even more likely to have dry mouth than tobacco users. Dry mouth is more common among young smokers, which suggests that we should perhaps pay more attention to raising awareness of the risks of smoking among young people and take appropriate measures to aid cessation. Because dry mouth can negatively impact people’s quality of life, oral hygienists should give more emphasis to tobacco and e-cigarette use in clinical practice.

The alluvian diagram showed that xerostomia was the bridge between the studied EHCs, supporting the theory that this manifestation is experienced not only by patients who have only HCV but also by those who present different EHMs. Crucially, the diagram shows no flow representing the co-occurrence of all three conditions (OLP, xerostomia, and SS-like) simultaneously in our cohort. Similarly, there was no co-occurrence observed for OLP and SS-like without the presence of xerostomia. Notably, 13.2% patients had both xerostomia and SS-like, (5 out of 6 patients with SS-like (83.33%)), while 18.4% of patients in the study group had OLP and xerostomia, (7 out of 15 patients with OLP (46.7%)). Considering that xerostomia can be a debilitating condition, once a diagnosis has been made and an underlying etiology has been identified, there are many therapeutic options for management that could help to alleviate the clinical manifestations of xerostomia [9,22].

The presence of OLP in our patients with HCV was also increased, with more than one-third of participants exhibiting this manifestation. A significant body of research has explored the association between OLP and HCV infection, leading to new insights into pathogenesis and treatment, with varying prevalence rates reported globally. The global association of HCV with OLP is reported to be variable. Recent data indicate that OLP patients have a four-fold higher frequency of HCV compared to controls with geographical variability. The African and Southeast Asian regions showed the highest odds ratios. In contrast, studies from the European region did not demonstrate a significant association. On a country level, Iraq and Egypt exhibited nearly ten-fold increases in risk, highlighting significant regional variations and possible immunogenetic influences, such as different HCV genotypes and human leukocyte antigens (HLAs) [4,8]. Although a causative role of chronic HCV infection in OLP occurrence has not yet been definitively elucidated, the OLP lesions’ improvement after viral eradication strongly supports a possible pathogenetic role of HCV chronic inflammation in the clinical course of OLP. These data could be crucial for the management of OLP, especially in countries where HCV is endemic [23].

Scientific literature revealed correlations that exist between numerous systemic diseases and HC, like diabetes and metabolic syndrome. The increased incidence of diabetes in patients with HCV may be associated with significant changes in the oral cavity. HCV may act as an independent diabetogenic factor [24]. We used the presence of other systemic diseases as exclusion criteria in order to avoid bias and this led to a reduction in the number of patients. This is a limitation of our study, but it gives solidity to our results. Further studies which also consider the connection of these EHC manifestations with other diseases, especially metabolic syndrome, are necessary.

There are studies that consider periodontal disease as an oral manifestation of HC mediated through both direct viral effects on oral tissues and indirect effects related to the immune response to HCV [4,25]. Even if it was not our aim to evaluate the periodontal status, we assessed the oral hygiene habits that are considered risk factors for periodontal disease. There are data in the literature that focus on the importance of maintaining oral hygiene in patients with OLP, xerostomia, or Sjogren syndrome [26,27,28]. The authors showed that structured plaque control intervention was effective in improving the oral health-related quality of life and clinically observed lesions in patients with OLP [26] and motivating patients to remove plaque led to a better quality of health and a significant improvement in their gingival lichen planus severity at 20 weeks compared to that observed in a control group that did not receive such advice [27]. A recent study by Stankeviciene et al. [28] showed that in patients with dry mouth, oral hygiene behaviors were suboptimal and needed to be improved; 67% of them reported not having a dental visit within the last year [28]. The review of Attin et Hornecker shows that there is consensus in the literature that (meticulous) tooth brushing once per day is sufficient to maintain oral health and to prevent periodontal diseases. However, most patients are not able to achieve sufficient plaque removal by performing oral hygiene measures at home. Therefore, tooth brushing twice daily is recommended by most dentists in order to improve plaque control and effectively maintain oral health [29]. In our study, daily teeth brushing was most prevalent habit (57.9%) and in terms of professional hygiene, the most frequent behavior was at 1 year (60.5%). The hygiene habit with the lowest percentage was twice daily brushing in patients with EHM, suggesting the importance of improving the frequency of oral hygiene practices in patients with HCV.

The statistical analysis revealed no association between these behaviors and the assessed EHM, but the low prevalence of a twice daily oral hygiene habit and the result of logistic regression regarding the importance of cigarettes number in xerostomia showed the necessity of having good control of these risk factors in patients with HCV [30,31].

The reduced number of patients resulting from the application of the exclusion criteria is a limitation of our study, contributing to the 76% power achieved and wide confidence intervals, limiting the ability to detect more significant association between variables and preventing us from dividing the patients into subgroups according to different parameters. Studies involving a larger group of patients in a multicenter study and analyses based on more parameters, like ratings of xerostomia severity or measurements of saliva flow, the type of OLP, or its oral distribution, etc., are needed.

## 5. Conclusions

Within the limits of this study, it could be suggested that oral hygiene and smoking could be considered risk factors for oral EHM of hepatitis C, and that controlling them is important for the quality of life of these patients. Gender has also been shown to be a risk factor for these manifestations.

## Figures and Tables

**Table 1:** Characteristics of the group.

Characteristics	Frequency N = 38
Age, years mean ± standard deviation median (interquartile range) range	56.47 ± 8.6 59.5 (52.5–63) 38–67
Gender, male	22 (57.9%)
Frequency of Dental Brushing	
Less than daily	7 (18.4%)
Daily	22 (57.9%)
Twice daily	9 (23.7%)
Professional Dental Hygiene Visits	
Less than 6 months	6 (15.8%)
At 1 year	23 (60.5%)
More than 1 year	9 (23.7%)
Smoking	
No	10 (26.3%)
Less than 10 cigarettes/day	16 (42.1%)
More than 10 cigarettes/day	12 (31.6%)
OLP	15 (39.5%)
Xerostomia	18 (47.4%)
Sjögren syndrome-like	6 (15.8%)

**Table 2:** Assessed EHC manifestations and oral hygiene habits.

Predictors	Xerostomia	OLP	SS-like
	n (%)	n (%)	n (%)
Frequency of Dental Brushing			
Less daily	6 (33.33)	4 (26.66)	2 (33.33)
Daily	10 (55.55)	9 (60)	3 (50)
Twice daily	2 (11.11)	2 (13.33)	1 (16.66)
Professional Dental Hygiene Visits			
Less than 6 mo	6 (33.33)	5 (33.33)	3 (50)
1 year	8 (44.44)	9 (60)	2 (33.33)
More 1 y	4 (22.22)	1 (6.66)	1 (16.66)

n—number of patients.

**Table 3:** Logistic regression analysis of factors associated with OLP.

Predictors	VIF (Multicollinearity)	OR (95% CI)	*p*-Value
Gender (female vs. male)	1.354	10.61 (1.78–94.45)	0.0086
Age	1.518	0.95 (0.84–1.06)	0.3401
Frequency of Dental Brushing			
Daily vs. less than daily	4.59	3.19 (0.1–118.5)	0.5028
Twice daily vs. less than daily	4.24	2.56 (0.04–247.1)	0.66
Professional Dental Hygiene Visits			
1 year vs. less than 6 mo	4.10	1.79 (0.02–211.4)	0.7975
More than 1 y vs. less than 6 mo	2.05	3.07 (0.23–93.36)	0.4148
Smoking			
Less 10 vs. More 10	3.23	0.24 (0.007–4.73)	0.344
No vs. More 10	2.44	0.15 (0.004–2.72)	0.211

OR—odds ratio; 95% CI—95% confidence intervals.

**Table 4:** Logistic regression analysis of factors associated with xerostomia.

Predictors	VIF (Multicollinearity)	OR (95% CI)	*p*-Value
Gender (female vs. male)	1.354	2.18 (0.43–12.17)	0.0345
Age	1.518	0.99 (0.89–1.12)	0.989
Frequency of Dental Brushing Daily vs. less than daily Twice daily vs. less than daily	4.59 4.24	0.2 (0.004–4.96) 0.09 (0.001–3.46)	0.331 0.21
Professional Dental Hygiene Visits 1 year vs. less than 6 mo More than 1 y vs. less than 6 mo	4.102 2.045	0.97 (0.03–30.29) 0.37 (0.04–2.89)	0.99 0.34
Smoking Less 10 vs. More 10 No vs. More 10	3.23 2.44	1.73 (0.14–28.14) 0.67 (0.05–10.60)	0.057 0.36

**Table 5:** Logistic regression analysis of factors associated with Sjögren syndrome-like.

Predictors	VIF (Multicollinearity)	OR (95% CI)	*p*-Value
Gender (female vs. male)	1.204	0.49 (0.05–4.23)	0.515
Age	1.286	1.101 (0.98–1.26)	0.103
Frequency of Dental Brushing			
Daily vs. less than daily	2.323	0.948 (0.068–10.58)	0.966
Twice daily vs. less than daily	2.254	1.094 (0.042–38.28)	0.956

## Data Availability

The original contributions presented in this study are included in the article. Further inquiries can be directed to the corresponding authors.
